# Supplementary material for: FERN – a Java framework for stochastic simulation and evaluation of reaction networks
Source: BMC Bioinformatics. 2008 Aug 29;9:356. doi: 10.1186/1471-2105-9-356 (PMC2553347; doi:10.1186/1471-2105-9-356)
Supplement: Additional file 1 — FERN distribution, Version 1.3. This archive contains the FERN source code and binaries as well as documentation and example models in FernML and SBML. [file 1471-2105-9-356-S1.zip › fern/doc/javadoc/fern/tools/gnuplot/CollectionAxes.html]

CollectionAxes


---


|  |  |  |
| --- | --- | --- |
| **Overview** \| **Package** \| **Class** \| **Use** \| **Tree** \| **Deprecated** \| **Index** \| **Help** | |  |
| **PREV CLASS**   **NEXT CLASS** | **FRAMES**    **NO FRAMES**     **All Classes** |  |
| SUMMARY: NESTED \| FIELD \| CONSTR \| METHOD | DETAIL: FIELD \| CONSTR \| METHOD |  |


---


## fern.tools.gnuplot Class CollectionAxes

```
java.lang.Object
  fern.tools.gnuplot.Axes
      fern.tools.gnuplot.CollectionAxes
```

**All Implemented Interfaces:**: Iterable<String>

---

``` public class CollectionAxes extends Axes ```

Implementation of `Axes` to use with `Collection`s or `Iterable`s
of any number type or array of numbers (the type of the collection is inferred by
using the reflection api).

**Author:**
:   Florian Erhard

**See Also:**: `Axes`

---

**Constructor Summary**

`CollectionAxes(Collection collection)`
  
          Create a new `Axes` object containing the given collection without labels /styles.

`CollectionAxes(Collection collection,
String[] labels,
String[] styles)`
  
          Create a new `Axes` object containing the given collection
with the given labels /styles (either can be `null`).

`CollectionAxes(Iterable iterable)`
  
          Create a new `Axes` object containing a collection (which is copied from
the given iterable) without labels /styles.

`CollectionAxes(Iterable iterable,
String[] labels,
String[] styles)`
  
          Create a new `Axes` object containing a collection (which is copied from
the given iterable) with the given labels /styles (either can be `null`).


| **Method Summary** | |
| --- | --- |
| `int` | `getNumColumns()`             Gets the number of columns. |
| `int` | `getNumRows()`             Gets the number of rows. |
| `Iterator<String>` | `iterator()`             Yields the tab separated columns row by row. |

**Methods inherited from class fern.tools.gnuplot.Axes**

`addAxes, applyDefaultStyle, getAdditionalAxesIterator, getLabel, getNumAdditionalColumns, getStyle, loadLabelsAndLineStyles, setLabel, setStyle`

**Methods inherited from class java.lang.Object**

`clone, equals, finalize, getClass, hashCode, notify, notifyAll, toString, wait, wait, wait`

**Constructor Detail**

### CollectionAxes

```
public CollectionAxes(Collection collection)
```

:   Create a new `Axes` object containing the given collection without labels /styles.

    **Parameters:**: `collection` - the collection

---


### CollectionAxes

```
public CollectionAxes(Iterable iterable)
```

:   Create a new `Axes` object containing a collection (which is copied from
    the given iterable) without labels /styles.

    **Parameters:**: `iterable` - the iterable

---


### CollectionAxes

```
public CollectionAxes(Collection collection,
                      String[] labels,
                      String[] styles)
```

:   Create a new `Axes` object containing the given collection
    with the given labels /styles (either can be `null`).

    **Parameters:**: `collection` - the collection: `labels` - labels for each column: `styles` - styles for each column

---


### CollectionAxes

```
public CollectionAxes(Iterable iterable,
                      String[] labels,
                      String[] styles)
```

:   Create a new `Axes` object containing a collection (which is copied from
    the given iterable) with the given labels /styles (either can be `null`).

    **Parameters:**: `iterable` - the iterable: `labels` - labels for each column: `styles` - styles for each column


**Method Detail**

### iterator

```
public Iterator<String> iterator()
```

:   Yields the tab separated columns row by row.

---


### getNumColumns

```
public int getNumColumns()
```

:   **Description copied from class: `Axes`**
:   Gets the number of columns. The number of the attached `Axes`
    object is also included.

    :   **Specified by:**: `getNumColumns` in class `Axes`
    :   **Returns:**: number of columns.

---


### getNumRows

```
public int getNumRows()
```

:   **Description copied from class: `Axes`**
:   Gets the number of rows.

    :   **Specified by:**: `getNumRows` in class `Axes`
    :   **Returns:**: number of rows.


---


|  |  |  |
| --- | --- | --- |
| **Overview** \| **Package** \| **Class** \| **Use** \| **Tree** \| **Deprecated** \| **Index** \| **Help** | |  |
| **PREV CLASS**   **NEXT CLASS** | **FRAMES**    **NO FRAMES**     **All Classes** |  |
| SUMMARY: NESTED \| FIELD \| CONSTR \| METHOD | DETAIL: FIELD \| CONSTR \| METHOD |  |


---
